# Differences in carbon source utilisation distinguish *Campylobacter jejuni* from *Campylobacter coli*

**DOI:** 10.1186/s12866-014-0262-y

**Published:** 2014-10-28

**Authors:** Sariqa Wagley, Jane Newcombe, Emma Laing, Emmanuel Yusuf, Christine M Sambles, David J Studholme, Roberto M La Ragione, Richard W Titball, Olivia L Champion

**Affiliations:** Biosciences, College of Life and Environmental Sciences, University of Exeter, Geoffrey Pope Building, Stocker Road, Exeter, EX4 4QD UK; Faculty of Health and Medical Sciences, School of Veterinary Medicine, University of Surrey, Guildford, Surrey GU2 7XH UK; Department of Bacteriology, Animal Health and Veterinary Laboratories Agency, Weybridge, Surrey KT15 3NB UK; Faculty of Health and Medical Sciences, School of Biosciences and Medicine, University of Surrey, Guildford, Surrey GU2 7XH UK

**Keywords:** *C. jejuni*, *C. coli*, Propanoate metabolism

## Abstract

**Background:**

*Campylobacter jejuni* and *C. coli* are human intestinal pathogens that are the most frequent causes of bacterial foodborne gastroenteritis in humans in the UK. In this study, we aimed to characterise the metabolic diversity of both *C. jejuni* and *C. coli* using a diverse panel of clinical strains isolated from the UK, Pakistan and Thailand, thereby representing both the developed and developing world. Our aim was to apply multi genome analysis and Biolog phenotyping to determine differences in carbon source utilisation by *C. jejuni* and *C. coli* strains.

**Results:**

We have identified a core set of carbon sources (utilised by all strains tested) and a set that are differentially utilised for a diverse panel of thirteen *C. jejuni* and two *C. coli* strains. This study used multi genome analysis to show that propionic acid is utilised only by *C. coli* strains tested. A broader PCR screen of 16 *C. coli* strains and 42 *C. jejuni* confirmed the absence of the genes needed for propanoate metabolism.

**Conclusions:**

From our analysis we have identified a phenotypic method and two genotypic methods based on propionic utilisation that might be applicable for distinguishing between *C. jejuni* and *C. coli*.

**Electronic supplementary material:**

The online version of this article (doi:10.1186/s12866-014-0262-y) contains supplementary material, which is available to authorized users.

## Background

*Campylobacter* species are a major cause of food-borne disease in the developed and the developing world. Over 80% of human cases are caused by *Campylobacter jejuni* and around 10% by *Campylobacter coli,* with the remaining human cases caused by other *Campylobacter* species. The species *C. jejuni* and *C. coli* are considered to be commensals of birds, domestic pets and livestock, but they have also been isolated from other sources including water, molluscs, bank voles and milk [1,2]. Disease in humans is most frequently associated with the consumption of contaminated poultry (*C. jejuni*) or porcine products (*C. coli*) [3].

Some past studies have begun to define the metabolic requirements of *C. jejuni*, [4-7], yet there is still a paucity of information regarding the metabolic flexibility of this bacterium, which is able to survive in such a wide range of environmental niches. It is well recognised that amino acids are important carbon and energy sources for *C. jejuni*, both *in vitro* and *in vivo* [8-11]. Serine, aspartic acid, glutamic acid and proline are the most common amino acids found in chicken faeces [4,12] and are therefore possible nutrient sources for *C. jejuni* in the chicken gut. *C. jejuni* preferentially metabolises amino acids in a specific order: serine, aspartatic acid, asparagine and then glutamic acid. Proline can also be metabolised, but only after other nutrients have been exhausted [13].

*Campylobacter* species were considered to be asaccharolytic and thus unable to metabolise glucose or other carbohydrates due to the absence of the glycolytic enzyme phosphofructokinase of the Embden-Meyerhof-Parnas (EMP) pathway and incomplete pentose phosphate (PPP) and Entner Doudoroff (ED) pathways [10,14]. However, it was recently shown that a novel L-fucose pathway including L-fucose permease is present within a genomic island in certain strains of *C. jejuni* [4,15]. These studies demonstrated that some *C. jejuni* strains can metabolise the sugar L-fucose. Furthermore, Stahl and co-workers found that fucose transport in *C. jejuni in vivo* provided these strains with a competitive advantage during colonisation of the piglet infection model [4]. However, there have been no subsequent reports of *C. jejuni* carbohydrate metabolism.

Citric acid cycle intermediates such as pyruvate, fumarate, oxaloacetate and 2-oxoglutarate, succinate, fumarate and malate have been reported as energy sources for *C. jejuni* [6,10,16]. In addition, *C. jejuni* has been reported to metabolise short-chain fatty acids (SCFA) [9,13]. Acetate, propionate and butyrate are the most significant anion or salt derivatives of SCFAs that are produced as metabolic by-products from the gut microbiota [17].

The capacity for *Campylobacter* species to survive and thrive in a wide range of environmental niches is underpinned by the ability of the bacteria to utilise the different metabolites that are available in the various hosts and environments. In contrast to the *C. jejuni* studies described above, very little is known about the metabolic requirements of *C. coli*. In this study, we aimed to characterise the metabolic profile of both *C. jejuni* and *C. coli* in order to see differences in their carbon utilisation capabilities. We used a diverse panel of clinical strains isolated from UK, Pakistan and Thailand, representing both the developed and developing world (Table 1). From our analysis we have identified a phenotypic method and two genotypic methods for distinguishing between *C. jejuni* and *C. coli*.

**Table 1:** **List of** ***C. jejuni*** **and** ***C. coli*** **strains used in this study**

**Species**	**Strain name**	**Source**	**Country of origin**	**2-methylcitrate synthase**	**Propionate-CoA ligase**	**gltA**	**Strain source**
*C. coli*	1/12	Human	UK	+	+	+	This study
*C. coli*	90-3	-	-	NT	NT	NT	AIMJ01000007.1
*C. coli*	2548	-	-	NT	NT	NT	AIML01000019.1
*C. coli*	JV 20	-	USA	NT	NT	NT	AEER01000027.1
*C. coli*	K3*	Human	Pakistan	+	+	+	This study
*C. coli*	K7*	Human	Pakistan	+	+	+	This study
*C. coli*	PS304*	Pig	UK	+	+	+	This study
*C. coli*	R03/167*	Cattle	UK	+	+	+	This study
*C. coli*	R03/389*	Sheep	UK	+	+	+	This study
*C. coli*	R03/393*	Cattle	UK	+	+	+	This study
*C. coli*	R04/28*	Sheep	UK	+	+	+	This study
*C. coli*	RM 2228	Chicken	USA	NT	NT	NT	[18]
*C. coli*	S03/35*	Pig	UK	+	+	+	This study
*C. coli*	S03/468*	Cattle	UK	+	+	+	This study
*C. coli*	S03/475*	Pig	UK	+	+	+	This study
*C. coli*	S03/704*	Pig	UK	+	+	+	This study
*C. coli*	S03/749*	Sheep	UK	+	+	+	This study
*C. coli*	S03/954*	Sheep	UK	+	+	+	This study
*C. coli*	S39-2-99-3*	Sheep	UK	+	+	+	This study
*C. coli*	Z163	-	-	NT	NT	NT	AIMK01000002.1
*C. coli*	MB17	Poultry	UK	+	+	+	This study
*C. jejuni*	74	Goose	-	-	-	+	This study
*C. jejuni*	95*	Goose	-	-	-	+	This study
*C. jejuni*	91B1	Chicken	-	-	-	+	This study
*C. jejuni*	99/201	Cow	UK	-	-	+	This study
*C. jejuni*	99/212	Human	UK	-	-	+	This study
*C. jejuni*	222*	Goose	-	-	-	+	This study
*C. jejuni*	11818	Human	UK	-	-	+	This study
*C. jejuni*	11919*	Human	UK	-	-	+	This study
*C. jejuni*	11974*	Human	UK	-	-	+	This study
*C. jejuni*	12241*	Human	UK	-	-	+	This study
*C. jejuni*	13305*	Human	UK	-	-	+	This study
*C. jejuni*	18629	Unknown	-	-	-	+	This study
*C. jejuni*	18836*	Unknown	-	-	-	+	This study
*C. jejuni*	30280*	Human	UK	-	-	+	This study
*C. jejuni*	30328*	Unknown	-	-	-	+	This study
*C. jejuni*	31407	Unknown	-	-	-	+	This study
*C. jejuni*	31481	Unknown	-	-	-	+	This study
*C. jejuni*	31484	Human	UK	-	-	+	This study
*C. jejuni*	31485	Human	UK	-	-	+	This study
*C. jejuni*	32787	Human	UK	-	-	+	This study
*C. jejuni*	32799*	Human	UK	-	-	+	This study
*C. jejuni*	33084*	Human	UK	-	-	+	This study
*C. jejuni*	33106	Human	UK	-	-	+	This study
*C. jejuni*	34007	Human	UK	-	-	+	This study
*C. jejuni*	40483	Unknown	-	-	-	+	This study
*C. jejuni*	41730	Unknown	-	-	-	+	This study
*C. jejuni*	44119*	Human	UK	-	-	+	This study
*C. jejuni*	47693*	Human	UK	-	-	+	This study
*C. jejuni*	81116*	Human	-	NT	NT	NT	[19]
*C. jejuni*	81176*	Human	USA	NT	NT	NT	[20]
*C. jejuni*	81205	Unknown	-	-	-	+	This study
*C. jejuni*	A6.T2.15*	Poultry	UK	-	-	+	This study
*C. jejuni*	A8.35.15A*	Poultry	UK	-	-	+	This study
*C. jejuni*	ATCC 43431	Human	-	-	-	+	[21]
*C. jejuni*	BB1267*	Human	UK	-	-	+	This study
*C. jejuni*	C1/C/2*	Poultry	UK	-	-	+	This study
*C. jejuni*	C5/T2/8	Poultry	UK	-	-	+	This study
*C. jejuni*	Cj1*	Human	Thailand	-	-	+	This study
*C. jejuni*	Cj2*	Human	Thailand	NT	NT	NT	This study
*C. jejuni*	Cj3*†	Human	Thailand	NT	NT	NT	This study
*C. jejuni*	Cj5*	Human	Thailand	-	-	+	This study
*C. jejuni*	D2/T/8	Poultry	UK	-	-	+	This study
*C. jejuni*	D2/T/95	Poultry	UK	-	-	+	This study
*C. jejuni*	K1*	Human	Pakistan	NT	NT	NT	This study
*C. jejuni*	K2*	Human	Pakistan	NT	NT	NT	This study
*C. jejuni*	K4*	Human	Pakistan	NT	NT	NT	This study
*C. jejuni*	K5*	Human	Pakistan	NT	NT	NT	This study
*C. jejuni*	K6*	Human	Pakistan	-	-	+	This study
*C. jejuni*	MB16	Poultry	UK	-	-	+	This study
*C. jejuni*	MB18	Poultry	UK	-	-	+	This study
*C. jejuni*	NCTC11168*	Human	-	-	-	+	[22]

^†^indicates those strains that have their genomes fully sequenced*. C. coli* strains K3 (AYKN00000000) and K7 (AYKO00000000) had their genomes sequenced in the scope of this study.

*indicates those strains used in the Biolog studies. NT indicates strains not tested.

## Results

### Carbon utilisation profiles of *C. jejuni* and *C. coli* strains reveal core and variable metabolites

We initially selected 13 human *C. jejuni* isolates and two human *C. coli* isolates to assess their metabolic profiles. Using BIOLOG PM1 and PM2 carbon utilisation plates, we identified the core (utilised by 100% of strains tested) and differential carbon sources (utilised by <90% of strains tested) utilised by a diverse panel of 13 *C. jejuni* and two *C. coli* strains (Table 2). The Omnilog software (Hayward, California USA) generates a reading every 15 mins corresponding to the conversion of the redox dye used in the assay and hence respiration levels. The entire experiment was repeated at least in triplicate for each strain. The mean values of the experimental replicates for each strain for each carbon substrate were compared by one way ANOVA and Tukey multiple comparison of means using a 95% family-wise confidence level. Statistical significance was assigned at the P < 0.05 level (see Additional file 1). Core carbon sources included the amino acids L-aspartic acid, L-asparagine, L-proline and L-serine. Citric acid cycle intermediate succinic acid and its derivative bromo-succinic acid were used by all strains. D,L malic acid, fumaric acid and methyl pyruvic acid were also metabolised by all of the strains. When D-malic acid was used as a sole carbon source it was only utilised by *C. jejuni* strains and not by the two *C. coli* strains tested. Lactic acid, butyric acid and D-lactic acid methyl ester were utilised by all strains while formic acid was utilised by 14 of the 15 strains tested.

**Table 2:** **Core and differential carbon sources utilised by strains used in this study**

	**Carbon source**	***C. jejuni***													***C. coli***	
		**K1**	**K2**	**K4**	**K5**	**K6**	**81176**	**81116**	**11168**	**32799**	**33084**	**Cj1**	**Cj2**	**Cj5**	**K3**	**K7**
Amino Acids	**L-Proline**	**+**	**+**	**+**	**+**	**+**	**+**	**+**	**+**	**+**	**+**	**+**	**+**	**+**	**+**	**+**
	**L-Asparagine**	**+**	**+**	**+**	**+**	**+**	**+**	**+**	**+**	**+**	**+**	**+**	**+**	**+**	**+**	**+**
	**L-Serine**	**+**	**+**	**+**	**+**	**+**	**+**	**+**	**+**	**+**	**+**	**+**	**+**	**+**	**+**	**+**
	**L-Aspartic Acid**	**+**	**+**	**+**	**+**	**+**	**+**	**+**	**+**	**+**	**+**	**+**	**+**	**+**	**+**	**+**
	L-Glutamic Acid	+	+	+	+	+	+	-	+	+	-	+	+	-	+	+
	L-Glutamine	+	+	+	+	+	+	+	+	+	-	+	+	-	+	+
Citric Acid Intermediates	**Methyl Pyruvate**	**+**	**+**	**+**	**+**	**+**	**+**	**+**	**+**	**+**	**+**	**+**	**+**	**+**	**+**	**+**
	**L-Malic Acid**	**+**	**+**	**+**	**+**	**+**	**+**	**+**	**+**	**+**	**+**	**+**	**+**	**+**	**+**	**+**
	**Succinic Acid**	**+**	**+**	**+**	**+**	**+**	**+**	**+**	**+**	**+**	**+**	**+**	**+**	**+**	**+**	**+**
	**D,L-Malic Acid**	**+**	**+**	**+**	**+**	**+**	**+**	**+**	**+**	**+**	**+**	**+**	**+**	**+**	**+**	**+**
	**Bromo Succinic Acid**	**+**	**+**	**+**	**+**	**+**	**+**	**+**	**+**	**+**	**+**	**+**	**+**	**+**	**+**	**+**
	**Fumaric Acid**	**+**	**+**	**+**	**+**	**+**	**+**	**+**	**+**	**+**	**+**	**+**	**+**	**+**	**+**	**+**
	Mono Methyl Succinate	**+**	**+**	**+**	**+**	**+**	**+**	**+**	**-**	**+**	**+**	**+**	**-**	**+**	**+**	**+**
	D-Malic Acid	**+**	**+**	**+**	**+**	**+**	**+**	**+**	**+**	**+**	**+**	**+**	**+**	**+**	**-**	**-**
Carboxylic Acids	**L-Lactic Acid**	**+**	**+**	**+**	**+**	**+**	**+**	**+**	**+**	**+**	**+**	**+**	**+**	**+**	**+**	**+**
	**D-Lactic Acid Methyl Ester**	**+**	**+**	**+**	**+**	**+**	**+**	**+**	**+**	**+**	**+**	**+**	**+**	**+**	**+**	**+**
	**a-Hydroxy Butyric Acid**	**+**	**+**	**+**	**+**	**+**	**+**	**+**	**+**	**+**	**+**	**+**	**+**	**+**	**+**	**+**
	Formic Acid	**+**	**+**	**+**	**+**	**+**	**+**	**+**	**+**	**-**	**+**	**+**	**+**	**+**	**+**	**+**
	Propionic Acid	-	-	-	-	-	-	-	-	-	-	-	-	-	+	+
Carbohydrates	L-Fucose	-	-	-	-	-	-	-	-	+	-	+	-	-	+	+

The carbon sources that are core to all the strains have been highlighted in bold.

Differential carbon sources (utilised by <90% of tested strains) included the carbohydrate L-fucose, supporting recent reports that some strains of *C. jejuni* are able to utilise carbohydrates [4]. We found differential metabolism of the amino acids L-glutamine, glycyl-L-glutamic acid and glycine-L-proline and citric acid cycle intermediates alpha-keto-glutaric acid, citric acid, glycolic acid, glyoxylic acid, tricarballyic acid. The metabolic profiles of all the strains can be found in Additional file 1. Finally, the SCFA propionic acid, was metabolised by both *C. coli* strains tested (K3 and K7) but was not metabolised by any of the *C. jejuni* strains (Table 2).

### Whole-genome sequencing reveals genes encoding propanoate metabolism enzymes in *C. coli* that are absent from *C. jejuni*

**Figure 1:**
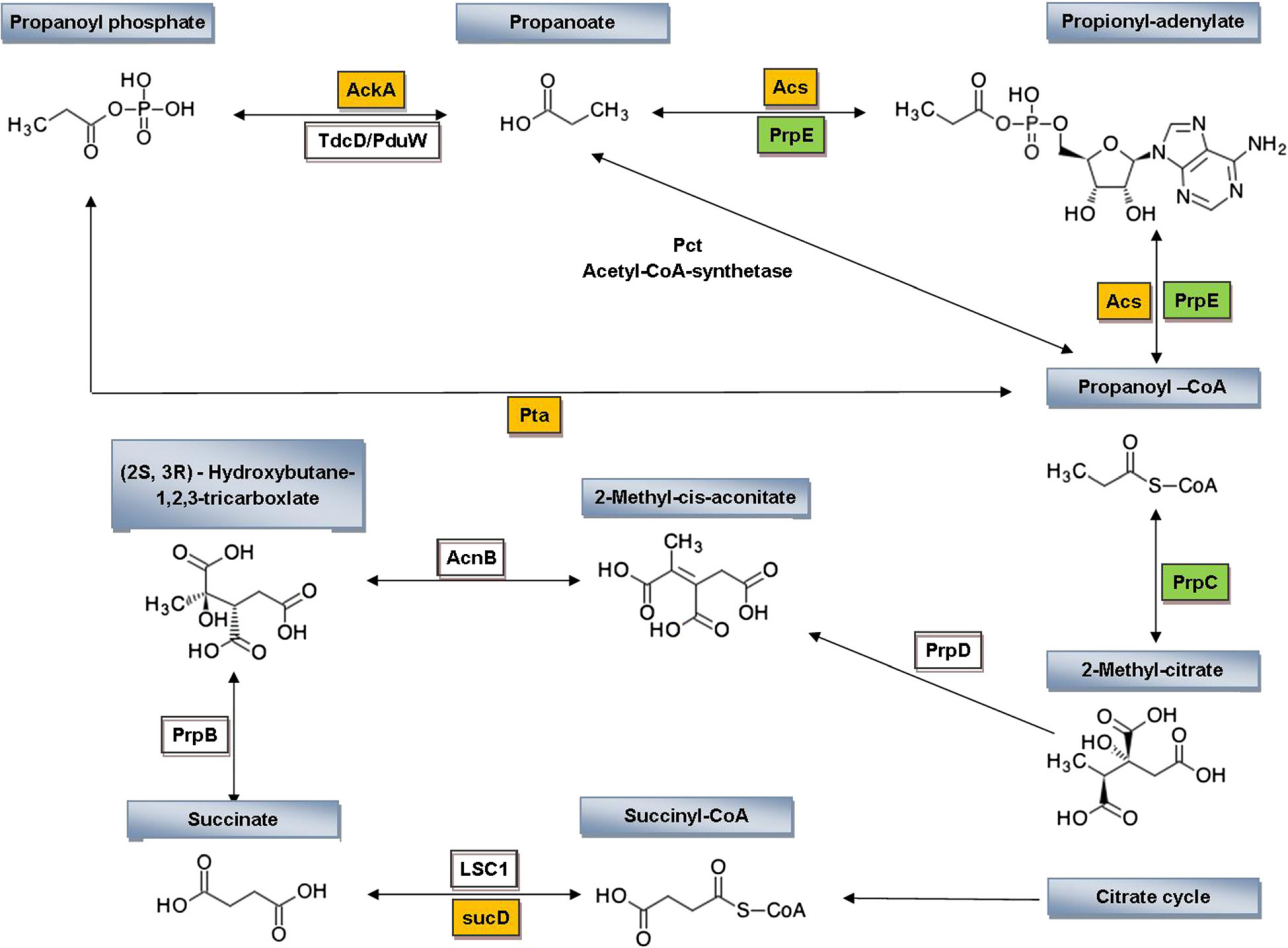
**Propanoate metabolism pathway in** ***C. jejuni*** **and** ***C. coli*** **.** Each box stands for an enzyme in the propanoate metabolism pathway. The yellow boxes indicate the ortholog genes shared by both *C. jejuni* and *C. coli* (ptA-phosphate acetyltrasferase, AckA-acetate kinase, AcS-acetyl-CoA synthetase and SucD- succinyl-CoA synthetase) while the green boxes indicates those genes present only in *C. coli* strains (PrpE- propionate-CoA ligase and PrpC-2-methyl citrate synthase).

To identify the genetic basis for propionic acid metabolism in *C. coli,* we sequenced the genomes of two *C. coli* strains (isolated from individuals suffering from diarrhoeal disease in Pakistan (Table 1). Data from these whole-genome shotgun projects have been deposited at DDBJ/EMBL/GenBank under the accessions AYKO00000000 and AYKN00000000. The versions described in this paper are version AYKO01000000 and AYKN00000000. We used the RAST webserver [23] to examine the presence or absence of enzymes in the propanoate metabolism pathway (KEGG: ko00640) for *C. jejuni* strains NCTC 11168 [22], 81116 [19] and 81176 [20] and *C. coli* strains K3 and K7 and found that genes encoding propionate-CoA ligase (PrpE/EC 6.2.1.17) and 2-methyl-citrate synthase (PrpC/EC 2.3.3.5) were present in the genomes of both *C. coli* strains but were absent from the *C. jejuni* genome sequences (see Figure 1).

To assess whether the presence or absence of propionate-CoA ligase and 2-methyl-citrate synthase genes could be used to distinguish *C. coli* from *C. jejuni*, we next searched the NCBI non-redundant nucleotide sequence database [24] for similar sequences using Blastn [25]. We also performed these searches against the 66 publicly available complete and draft *C. coli* genome sequences in RefSeq [26]. Homologues of propionate-CoA ligase and 2-methyl-citrate synthase genes were identified in all (66/66) of the *C. coli* genomes interrogated and with a minimum of 99% identity across the entire gene sequences. In contrast, when the 84 publicly available *C. jejuni* complete and draft genomes were interrogated we did not identify genes that could encode propionate-CoA ligase or 2-methyl-citrate synthase.

### A PCR assay for the propionate-CoA ligase or 2 methyl-citrate-synthase genes can be used to distinguish *C. coli* from *C. jejuni*

An amino acid sequence alignment of propionate-CoA ligase and 2-methyl-citrate-synthase from *C. coli* revealed that these proteins are highly conserved across six randomly selected *C. coli* isolates (see Additional file 2). PCR amplification of the propionate-CoA ligase or 2-methyl-citrate synthase genes using *C. coli* or *C. jejuni* template DNA generated amplicons of the expected size from all 15 strains of *C. coli* tested. In contrast, amplicons were not generated using template DNA from 42 of 43 strains of *C. jejuni* tested. The single isolate (MB17 previously characterised as *C. jejuni*) that generated PCR amplicons was subsequently shown to give a negative reaction in the hippurate hydrolysis assay, indicating that this strain had been incorrectly assigned as *C. jejuni*. Therefore, 100% (42/42) of the *C. jejuni* strains failed to yield amplicons. Positive-control PCR assays for the housekeeping gene *gltA* (encoding citrate synthase) generated amplicons from all of the *C. jejuni* and *C. coli* isolates tested.

### Propionic acid as a sole carbon source allows differential identification of *C. coli* and *C. jejuni*

**Figure 2:**
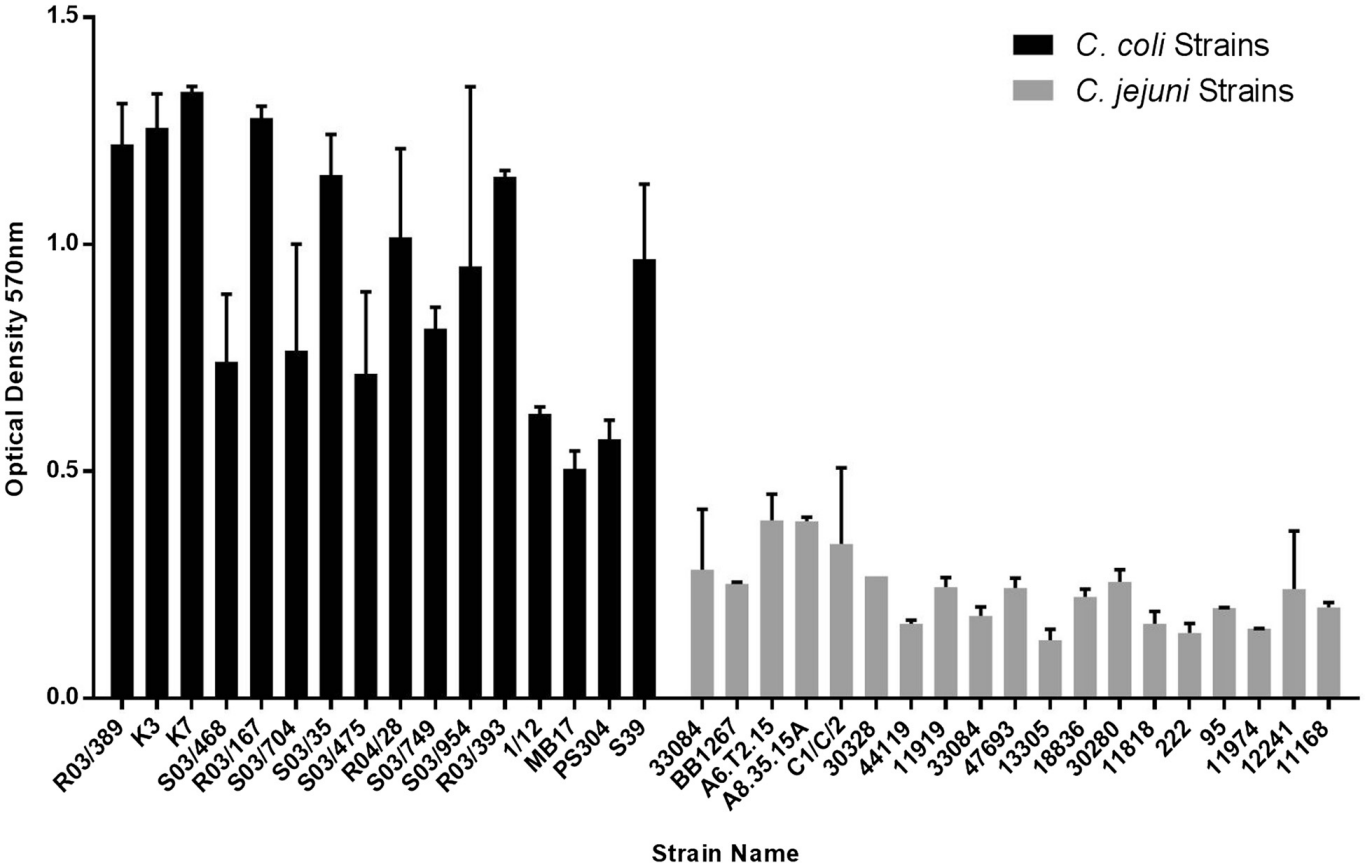
**Histogram representing propionic utilisation by** ***C. jejuni*** **and** ***C. coli*** **strains.** The *C. jejuni* strains have been grouped together in grey bars while all *C. coli* strains have been grouped by black bars. No or only weak propionic utilisation was seen in all *C. jejuni* strains tested (values below an OD of 0.4) while high propionic utilisation was seen for all *C. coli* strains tested (values above an OD of 0.5).

To determine whether *C. coli* and *C. jejuni* differed in their ability to utilise propionic acid as a sole carbon source in culture medium*,* we tested a panel of 16 environmental and clinical *C. coli* strains and 17 *C. jejuni* strains in BIOLOG phenotypic microarray plates containing propionic acid as the sole carbon source. No or weak propionic utilisation was seen in all *C. jejuni* strains tested while strong propionic utilisation was seen for all *C. coli* strains tested. The mean values of the experimental replicates for each strain were compared using a unpaired *T* test with Welch’s correction. Statistical significance was assigned at P < 0.0001 level indicating a difference between propionic utilisations by *C. jejuni* and *C. coli*. Figure 2 shows levels of propionic acid utilisation by *C. jejuni* and *C. coli* strains.

## Discussion

*Campylobacter* species are fastidious bacteria, exhibiting significant metabolic restrictions compared to other enteropathogenic bacteria. The principal energy sources for *C. jejuni* are amino acids, citric acid cycle intermediates and short-chain fatty acids [10,27]. Despite these metabolic limitations, *Campylobacter* species can survive in a wide range of environmental niches suggesting metabolic flexibility. To date, only a few laboratory adapted strains have been used to study the metabolic diversity of *Campylobacter* species. In this study, we examined the extent of the metabolic diversity of *C. jejuni* and *C. coli* recently isolated from cases of disease.

The catabolism of amino acids by *C. jejuni* not only provides a carbon source but also protects bacteria from osmotic and oxidative stress [28-30]. Moreover, catabolism of particular amino acids can confer tissue-specific advantages for *C. jejuni* colonisation [11]. Hofreuter and co-workers showed that serine supports growth of *C. jejuni* in murine intestinal and liver tissue, whereas proline and glutamine support growth in the intestine but not the liver [11]. They also showed that two *C. jejuni* strains (ATCC 33251 and RM1221) were unable to utilise serine *in vitro* even though they showed no genetic variation in L-serine dehydratase (SdaA) and the serine transporter SdaC. We have shown in this study that L-serine was utilised by all 13 *C. jejuni* tested. Amino acids that were differentially utilised by the *C. jejuni* and *C. coli* strains included L-glutamine, and glutamic acid. This difference in the metabolic profiles of *C. jejuni* and *C. coli* isolates may indicate adaptation of these strains to different niches and reflect niche specific requirements for amino acids as a nutrient source, such as those reported for *Staphylococcus aureus* and *Francisella novicida* [31,32].

Previous studies have concluded that except for a few strains that are able to utilise the carbohydrate L-fucose [4,10], *Campylobacter* are asaccharolytic. One of the strains shown in previous studies [4,15] to utilise fucose was *C. jejuni* strain NCTC 11168. In our study, we found that strain NCTC 11168 was unable to respire using this carbon source. Other workers have reported differences in strains of NCTC 11168 which may account for this phenotype [33,34]. Furthermore, in a previous study it has been shown that oxidation of some substrates was greater at 42°C than 37°C. This may account for the lack of L-fucose utilisation seen here [35]. The ability of some strains of *C. jejuni* to utilise L-fucose is reportedly associated with the colonisation of pigs, but not poultry [4]. The two *C. coli* strains we tested using the BIOLOG assay were both able to utilise L-fucose. As *C. coli* is predominately found in pigs, our findings support the suggestion [4] that fucose availability in the pig intestine influences the bacterial fitness of *C. coli* colonisation in this host.

From our study, we found that none of the *C. jejuni* isolates tested were able to metabolise propionic acid while both *C. coli* strains were able to metabolise this carbon source. This appears to be linked to the presence of propanoate-CoA ligase and 2-methyl-synthase genes in *C. coli* and their absence in *C. jejuni*. Our studies further showed that the absence or presence of these genes is a reliable marker for the identification of *C. jejuni* or *C. coli*. The power of this test was confirmed by our finding that strain MB17 has been erroneously identified as *C. jejuni*; this strain tested positive for these two marker genes and our subsequent investigations showed that this was a strain of *C. coli*.

Propionic acid is the most abundant of four SCFA measured in pig manure [36] and levels of propionate have been reported to be high in the gastrointestinal tract of pigs, one of the natural hosts for *C. coli* [37]. Competition for nutrients in the gut is fierce and so the ability to use a potentially toxic metabolic waste product as an energy source may confer *C. coli* with a selective advantage when colonising the pig gastrointestinal tract.

Campylobacterioisis is the principal cause of bacterial diarrhoeal disease in humans and *C. jejuni* and *C. coli* are the most common aetiological agents of human campylobacteriosis. The epidemiology of *Campylobacter* infections in humans is poorly characterised in low income countries [38]. The true incidence of *C. coli* is difficult to estimate due to the difficulties in distinguishing *Campylobacter* species. *C. jejuni* can be distinguished from other *Campylobacter* species on the basis of the hydrolysis of hippurate for which *C. jejuni* gives only a positive reaction. However, hippurate negative *C. jejuni* strains have occasionally been reported [39,40]. Mass spectrometry has also been used to discriminate between *Campylobacter* species *C. coli*, *C. jejuni*, *C. helveticus*, *C. lari*, *C. sputorum* and *C. upsaliensis* using species identifying biomarkers [41]. However, these methods are time consuming and require complex equipment. Often additional confirmatory tests based upon PCR amplification of a range of genes are needed [42-44]. For example Klena et al.*,* describes a multiplex PCR test to distinguish between *C. jejuni, C. coli, C. coli, C. lari* and *C. upsaliensis* using the lipid A gene *lpxA* with good species identification.

## Conclusion

Methods that distinguish species of *Campylobacter* need to be developed in order to understand environmental aspects of *Campylobacter* epidemiology, the emergence of atypical *Campylobacter* species, emerging clinical aspects of disease, the association of *Campylobacter* species with their natural hosts and emerging trends in antibiotic resistance. We have presented a molecular method that allows for accurate discrimination of the two predominant species associated with human disease. In addition, we have identified the basis for a selective media that would permit rapid discrimination of *C. jejuni* and *C. coli* that would be suitable for use in diagnostic and reference laboratories around the world. The importance of identifying *C. coli* from *C. jejuni* correctly from both environmental and clinical sources would help broaden our understanding of the distribution, prevalence, geographical spread and transmission of this human pathogen.

## Methods

### Strains and culture conditions

All bacterial strains used in this study are shown in Table 1. *Campylobacter* strains were cultured on Mueller Hinton Agar (Oxoid, UK), Columbia agar plates (CBA) (Oxoid, UK) supplemented with either 5% (v/v) horse blood or with Skirrow selective supplement (Oxoid, UK) in a variable atmosphere incubator (VAIN) (Don Whitley Scientific, UK) under microaerobic conditions (5% O_2_, 85% N_2_, 10% CO_2_) at 37°C for 24 or 48 h or alternatively microaerophillic conditions were created using CampyGen Paks (Fisher Ltd).

### Genome sequencing, assembly and annotation

Bacterial cells were harvested and genomic DNA was extracted using Wizard Genomic DNA Purification Kit (Promega A1125). Each gDNA sample was quantified using a Thermo Scientific NanoDrop™ 1000 Spectrophotometer and run on a 1.5% agarose gel stained with SYBR Safe DNA stain. Genome-wide sequence data was generated for *C. coli* K3 and K7 (Table 1) using the Illumina HiSeq 2500 platform. Sequencing reads were assembled *de novo* using Velvet [19] using hash lengths between 39 and 57 and coverage cut-offs between 2 and 10. Assemblies were submitted to the RAST online server [23] for annotation.

### Carbon utilisation phenotypic microarray assay

*C. jejuni* and *C. coli* isolates were cultured from (−80°C) frozen stocks on Columbia Blood Agar (CBA) containing 5% horse blood (HB) for 48 h at 37°C. Cultures were transferred onto Mueller Hinton agar and incubated as before for an additional 48 h. The bacteria were then harvested from agar plates and resuspended into IF-0a inoculating fluid (Biolog, Hayward, CA, USA) and adjusted to 16% transmittance (or using an optical density (OD) measuring device (Turbidimeter, Biolog) a OD_600_ = 0.8). The cell suspension was mixed with an additive solution containing tetrazolium violet (Redox dye D) as a redox indicator dye, PM1 or PM2A additive and water, as per the manufacturer’s instructions. A final volume of 100 μl was added to each of the 96 wells in the carbon utilisation plates, PM1 and PM2A, or bespoke propionic acid only PM plates (Biolog). Care was taken to carefully maintain microaerobic conditions at all times, and inoculated PM plates were placed into gas impermeable bags with a Campy Gen Compact Sachet (Oxoid, UK) during the assay. The inoculated PM1 and PM2A plates were immediately placed in an Omnilog automatic plate reader (Biolog) for incubation at 37°C. Metabolism of the various carbon sources was recorded every 15 min over a 90 h period as reduction of the tetrazolium violet dye, producing a purple colour, indicative of active bacterial respiration. The bespoke propionic plates were immediately placed in a variable atmosphere incubator (VAIN) (Don Whitley Scientific, UK) under microaerobic conditions (5% O_2_, 85% N_2_, 10% CO_2_) at 37°C for up to 5 days. Metabolism of propionic acid was recorded daily over five days as a reduction of the tetrazolium violet dye, producing a purple colour, indicative of active bacterial respiration. Abiotic negative control plates containing all components except for cells were also analysed. Initially, we performed the abiotic control experiments to identify carbon sources that could indicate in false positive results due to auto- reduction of the dye. False positive results observed in PM1 were L-arabinose, D-xylose, D-ribose, L-lyxose. Potential false positive results observed in PM2 were, D-arabinose, 2-deoxy D ribose, D glucosamine, 5-keto D gluconic acid, and dihyroxyacetone. Results for these carbon sources were subsequently excluded from further analysis.

### Bioinformatics analysis

The Kyoto Encyclopaedia of Gene and Genomes (KEGG (http://www.genome.jp/kegg) was employed to determine propanoate metabolism pathways to which the proteins were assigned. The RAST webserver [23] was used to automatically annotate the genome sequences and to carry out KEGG pathway comparisons. To search for conserved regions in the sequences of propionate-CoA ligase and 2-methyl citrate synthase, gene sequences from newly sequenced *C. coli* isolates K3 and K7 were aligned with homologues in *C. coli* genome sequences available through the National Center for Biotechnology Information (NCBI) website available at (http://www.ncbi.nlm.nih.gov/). Homology searches were performed using the Blastn tools [25] and multiple sequence alignments were carried out using the computer software Clone Manager 9 (Cary, North Carolina USA).

### Genetic screening for propionate-CoA ligase and 2 methyl citrate synthase

A 522 bp portion of the *propionate-CoA ligase* was amplified using the PCR from 43 strains of *C. jejuni* DNA and 14 *C. coli* DNA using the primers PA2 (5′- ATA GGG TGC TTG ATG ATA GCG ATG GG-3′ and 5′- GAA GCA TAT TTG CTC TAT ATT GTG GGC GTT-3′). A 843 bp portion of the *2 methyl citrate synthase* was amplified using the PCR from 43 strains of *C. jejuni* DNA and 14 *C. coli* DNA using the primers PA1 (5′ – TAA AAA AAC GGG TGG ATT GGC AGG AGT TAT-3′ and 5′- GTG CAT TTC TAG GAT CAC CTC CAA GTC-3′. A 250 bp portion of the internal control gene *gltA* was also amplified to check the fidelity of the PCR for all *Campylobacter* strains using the primers gltA (5′ - TAA AAT CCC TAC TAT AGT GGC CAC CG-3′ and 5′GCA TAA GGA TGA GCA TGA GTT GAA CC-3′). Amplifications were carried out in a 50 μl reaction mix containing 2-5 μl DNA and using Red Hot Start Polymerase (Qiagen Ltd) and then subjected to PCR using the following cycles; for PA1/PA2 96°C for 15 minutes, followed by 25 cycles of 1 minute at 94°C, 1.5 min at 59.4°C, 1.5 min at 72°C, and 7 min at 72°C; for *gltA* 15 min at 96°C, 30 cycles of 1 minute at 94°C, 1 minute at 59.4°C, 1.5 min at 72°C and 7 min at 72°C.

## Additional files

Additional file 1:
**Raw data on carbon utilisation of** ***C. jejuni*** **and** ***C.coli*** **strains using Biolog analysis.**
Additional file 2:
**Comparison of (a) 2-methyl citrate synthase and (b) propionate-CoA ligase amino acid sequences from** ***C. coli*** **strain.** The amino acid sequences were analysed by computer software Clone Manager Professional Suite version 9 (Cary, North Carolina USA). Dashed lines represent gaps generated by the analysis software. The identical amino acids are shaded in pale green and the strains can be identified by strain name. The amino acid sequence of 2-methyl citrate synthase and propionate-CoA ligase proteins can be accessed through the NCBI protein database under NCBI accession numbers AAFL01000003.1 (RM2228) [18], AEER01000027.1 (JV 20), AIMK01000002.1 (Z163), AIMJ01000007.1 (90–3), AIML01000019.1 (2548).
