# Supplementary material for: Differences in carbon source utilisation distinguish Campylobacter jejuni from Campylobacter coli
Source: BMC Microbiol. 2014 Oct 28;14:262. doi: 10.1186/s12866-014-0262-y (PMC4219013; doi:10.1186/s12866-014-0262-y)
Supplement: Additional file 2: — Comparison of (a) 2-methyl citrate synthase and (b) propionate-CoA ligase amino acid sequences from C. coli strain. The amino acid sequences were analysed by computer software Clone Manager Professional Suite version 9 (Cary, North Carolina USA). Dashed lines represent gaps generated by the analysis software. The identical amino acids are shaded in pale green and the strains can be identified by strain name. The amino acid sequence of 2-methyl citrate synthase and propionate-CoA ligase proteins can be accessed through the NCBI protein database under NCBI accession numbers AAFL01000003.1 (RM2228) [18], AEER01000027.1 (JV 20), AIMK01000002.1 (Z163), AIMJ01000007.1 (90–3), AIML01000019.1 (2548). [file 12866_2014_262_MOESM2_ESM.pdf]

A: 2-methyl citrate synthase

```
K7          1 msiseatkktgglagviaggqsaictcglgnglnyygysieelaqnaefeeiayllqfgelpnakelenykekiiaqralsenlknvlkaipkevhpmnlmqtavaalgalegenedfsdqdekiirllgilpsmlcywhhyvnfgkeidf
RM2228      1 msiseatkktgglagviaggqsaictcglgnglnyygysieelaqnaefeeiayllqfgelpnakelenykekiiaqralsenlknvlkaipkevhpmnlmqtavaalgalegenedfsdqdekiirllgilpsmlcywhhyvnfgkeidf
JV20        1 msiseatkktgglagviaggqsaictcglgnglnyygysieelaqnaefeeiayllqfgelpnakelenykekiiaqralsenlknvlkaipkevhpmnlmqtavaalgalegenedfsdqdekiirllgilpsmlcywhhyvnfgkeidf
Z163        1 msiseatkktgglagviaggqsaictcglgnglnyygysieelaqnaefeeiayllqfgelpnakelenykekiiaqralsenlknvlkaipkevhpmnlmqtavaalgalegenedfsdqdekiirllgilpsmlcywhhyvnfgkeidf
90-3        1 msiseatkktgglagviaggqsaictcglgnglnyygysieelaqnaefeeiayllqfgelpnakelenykekiiaqralsenlknvlkaipkevhpmnlmqtavaalgalegenedfsdqdekiirllgilpsmlcywhhyvnfgkeidf
2548        1 msiseatkktgglagviaggqsaictcglgnglnyygysieelaqnaefeeiayllqfgelpnakelenykekiiaqralsenlknvlkaipkevhpmnlmqtavaalgalegenedfsdqdekiirllgilpsmlcywhhyvnfgkeidf

K7        151 dsnqtsiagyfleklkleapkedfikamqcslilyaehefnastftaricastksdifsavaaaigalrgplhgganeaamhliesfksvedaiegvnkklenkellmgfghrvyglggdprnalikvwskhlggddllfkiseaiealm
RM2228    151 dsnqtsiagyfleklkleapkedfikamqcslilyaehefnastftaricastksdifsavaaaigalrgplhgganeaamhliesfksvedaiegvnkklenkellmgfghrvyglggdprnalikvwskhlggddllfkiseaiealm
JV20      151 dsnqtsiagyfleklkleapkedfikamqcslilyaehefnastftaricastksdifsavaaaigalrgplhgganeaamhliesfksvedaiegvnkklenkellmgfghrvyglggdprnalikvwskhlggddllfkiseaiealm
Z163      151 dsnqtsiagyfleklkleapkedfikamqcslilyaehefnastftaricastksdifsavaaaigalrgplhgganeaamhliesfksvedaiegvnkklenkellmgfghrvyglggdprnalikvwskhlggddllfkiseaiealm
90-3      151 dsnqtsiagyfleklkleapkedfikamqcslilyaehefnastftaricastksdifsavaaaigalrgplhgganeaamhliesfksvedaiegvnkklenkellmgfghrvyglggdprnalikvwskhlggddllfkiseaiealm
2548      151 dsnqtsiagyfleklkleapkedfikamqcslilyaehefnastftaricastksdifsavaaaigalrgplhgganeaamhliesfksvedaiegvnkklenkellmgfghrvyglggdprnalikvwskhlggddllfkiseaiealm

K7        301 kekrpslppnadfysasayhfmgipteyftpifimsrvsgwcahikeqrannklirpsseyigveprkfvkiqdr
RM2228    301 kekrpslppnadfysasayhfmgipteyftpifimsrvsgwcahikeqrannklirpsseyigveprkfvkiqdr
JV20      301 kekrpslppnadfysasayhfmgipteyftpifimsrvsgwcahikeqrannklirpsseyigveprkfvkiqdr
Z163      301 kekrpslppnadfysasayhfmgipteyftpifimsrvsgwcahikeqrannklirpsseyigveprkfvkiqdr
90-3      301 kekrpslppnadfysasayhfmgipteyftpifimsrvsgwcahikeqrannklirpsseyigveprkfvkiqdr
2548      301 kekrpslppnadfysasayhfmgipteyftpifimsrvsgwcahikeqrannklirpsseyigveprkfvkiqdr
```

B: propionate CoA ligase

```
K7          1 -----------mgivyeqtyreslqnpeafwaeaakkvhwynewdrvlddsdghyrwfvggcmntcynaldlhvhngrgdqlaliydspvtdtkkkytykqlrdrvakvagilankgvvkgdrvviympmipealiamlacarlgaihsv
RM2228      1 -----------mgivyeqtyreslqnpeafwaeaakkvhwynewdrvlddsdghyrwfvggcmntcynaldlhvhngrgdqlaliydspvtdtkkkytykqlrdrvakvagilankgvvkgdrvviympmipealiamlacarlgaihsv
90-3        1 -----------mgivyeqtyreslqnpeafwaeaakkvhwynewdrvlddsdghyrwfvggcmntcynaldlhvhngrgdqlaliydspvtdtkkkytykqlrdrvakvagilankgvvkgdrvviympmipealiamlacarlgaihsv
2548        1 -----------mgivyeqtyreslqnpeafwaeaakkvhwynewdrvlddsdghyrwfvggcmntcynaldlhvhngrgdqlaliydspvtdtkkkytykqlrdrvakvagilankgvvkgdrvviympmipealiamlacarlgaihsv
JV20        1 mqiqlikkgwimgivyeqtyreslqnpeafwaeaakkvhwynewdrvlddsdghyrwfvggcmntcynaldlhvhngrgdqlaliydspvtdtkkkytykqlrdrvakvagilankgvvkgdrvviympmipealiamlacarlgaihsv
Z163        1 -----------mgivyeqtyreslrnpeafwaeaakkvhwynewdrvlddsdghyrwfvggcmntcynaldlhvhngrgdqlaliydspvtdtkkkytykqlrdrvakvagilankgvvkgdrvviympmipealiamlacarlgaihsv

K7        140 vfggfaahelatriedakprmiisascgievssiieykpildeaikkcthkpttcliwqrpqyranmlpwrdidweleeektrgvdpvpvlatdplyilytsgttgspkgvirsngghsvamkwsmdniynakagdvfftasdvgwvvgh
RM2228    140 vfggfaahelatriedakprmiisascgievssiieykpildeaikkcthkpttcliwqrpqyranmlpwrdidweleeektrgvdpvpvlatdplyilytsgttgspkgvirsngghsvamkwsmdniynakagdvfftasdvgwvvgh
90-3      140 vfggfaahelatriedakprmiisascgievssiieykpildeaikkcthkpttcliwqrpqyranmlpwrdidweleeektrgvdpvpvlatdplyilytsgttgspkgvirsngghsvamkwsmdniynakagdvfftasdvgwvvgh
2548      140 vfggfaahelatriedakprmiisascgievssiieykpildeaikkcthkpttcliwqrpqyranmlpwrdidweleeektrgvdpvpvlatdplyilytsgttgspkgvirsngghsvamkwsmdniynakagdvfftasdvgwvvgh
JV20      151 vfggfaahelatriedakprmiisascgievssiieykpildeaikkcthkpttcliwqrpqyranmlpwrdidweleeektrgvdpvpvlatdplyilytsgttgspkgvirsngghsvamkwsmdniynakagdvfftasdvgwvvgh
Z163      140 vfggfaahelatriedakprmiisascgievssiieykpildeaikkcthkpttcliwqrpqyranmlpwrdidweleeektrgvdpvpvlatdplyilytsgttgspkgvirsngghsvamkwsmdniynakagdvfftasdvgwvvgh

K7        290 syivyaplmngcttivyegkpvrtpnpsafwriieeykvnvlfsaptafraikkedpkgewikkfnldslrsifvagercdsdtlkwiekltkkpvidnwwqtetgwaiaanplgleaqvvkagsptkpmpgfnlkvldekgqelgakk
RM2228    290 syivyaplmngcttivyegkpvrtpnpsafwriieeykvnvlfsaptafraikkedpkgewikkfnldslrsifvagercdsdtlkwiekltkkpvidnwwqtetgwaiaanplgleaqvvkagsptkpmpgfnlkvldekgqelgakk
90-3      290 syivyaplmngcttivyegkpvrtpnpsafwriieeykvnvlfsaptafraikkedpkgewikkfnldslrsifvagercdsdtlkwiekltkkpvidnwwqtetgwaiaanplgleaqvvkagsptkpmpgfnlkvldekgqelgakk
2548      290 syivyaplmngcttivyegkpvrtpnpsafwriieeykvnvlfsaptafraikkedpkgewikkfnldslrsifvagercdsdtlkwiekltkkpvidnwwqtetgwaiaanplgleaqvvkagsptkpmpgfnlkvldekgqelgakk
JV20      301 syivyaplmngcttivyegkpvrtpnpsafwriieeykvnvlfsaptafraikkedpkgewikkfnldslrsifvagercdsdtlkwiekltkkpvidnwwqtetgwaiaanplgleaqvvkagsptkpmpgfnlkvldekgqelgakk
Z163      290 syivyaplmngcttivyegkpvrtpnpsafwriieeykvnvlfsaptafraikkedpkgewikkfnldslrsifvagercdsdtlkwiekltkkpvidnwwqtetgwaiaanplgleaqvvkagsptkpmpgfnlkvldekgqelgakk

K7        440 gilclklplppaclmgiwenderyrrgyldqfpgyyltgdtgyidkdgyvyvlgrmdgiinvaghrlstgemeeiiakhpdvaecavigvndelkgeipmgfivlkegierdhrgivegvvalvrqeigavasfkiatvvsalpktrsgk
RM2228    440 gilclklplppaclmgiwenderyrrgyldqfpgyyltgdtgyidkdgyvyvlgrmdgiinvaghrlstgemeeiiakhpdvaecavigvndelkgeipmgfivlkegierdhrgivegvvalvrqeigavasfkiatvvsalpktrsgk
90-3      440 gilclklplppaclmgiwenderyrrgyldqfpgyyltgdtgyidkdgyvyvlgrmdgiinvaghrlstgemeeiiakhpdvaecavigvndelkgeipmgfivlkegierdhrgivegvvalvrqeigavasfkiatvvsalpktrsgk
2548      440 gilclklplppaclmgiwenderyrrgyldqfpgyyltgdtgyidkdgyvyvlgrmdgiinvaghrlstgemeeiiakhpdvaecavigvndelkgeipmgfivlkegierdhrgivegvvalvrqeigavasfkiatvvsalpktrsgk
JV20      451 gilclklplppaclmgiwenderyrrgyldqfpgyyltgdtgyidkdgyvyvlgrmdgiinvaghrlstgemeeiiakhpdvaecavigvndelkgeipmgfivlkegierdhrgivegvvalvrqeigavasfkiatvvsalpktrsgk
Z163      440 gilclklplppaclmgiwenderyrrgyldqfpgyyltgdtgyidkdgyvyvlgrmdgiinvaghrlstgemeeiiakhpdvaecavigvndelkgeipmgfivlkegierdhrgivegvvalvrqeigavasfkiatvvsalpktrsgk

K7        590 ilrknlreiadgstlnvpatiedsnvlkacekainalgypknkgek
RM2228    590 ilrknlreiadgstlnvpatiedsnvlkacekainalgypknkgek
90-3      590 ilrknlreiadgstlnvpatiedsnvlkacekainalgypknkgek
2548      590 ilrknlreiadgstlnvpatiedsnvlkacekainalgypknkgek
JV20      601 ilrknlreiadgstlnvpatiedsnvlkacekainalgypknkgek
Z163      590 ilrknlreiadgstlnvpatiedsnvlkacekainalgypknkgek
```
